# Immunoglobulins: 25 Years of Immunoinformatics and IMGT-ONTOLOGY

**DOI:** 10.3390/biom4041102

**Published:** 2014-12-16

**Authors:** Marie-Paule Lefranc

**Affiliations:** IMGT^®^, the international ImMunoGenetics information system^®^, Laboratoire d’ImmunoGénétique Moléculaire LIGM, Institut de Génétique Humaine IGH, UPR CNRS 1142, Montpellier University, 141 rue de la Cardonille, 34396 Montpellier cedex 5, France; E-Mail: Marie-Paule.Lefranc@igh.cnrs.fr; Tel.: +33-4-34-35-99-65; Fax: +33-4-34-35-99-01

**Keywords:** IMGT, immunogenetics, immunoinformatics, IMGT-ONTOLOGY, IMGT Collier de Perles, immunoglobulin, immune repertoire, IMGT unique numbering, next generation sequencing, antibody humanization

## Abstract

IMGT^®^, the international ImMunoGeneTics information system^®^ (CNRS and Montpellier University) is the global reference in immunogenetics and immunoinformatics. By its creation in 1989, IMGT^®^ marked the advent of immunoinformatics, which emerged at the interface between immunogenetics and bioinformatics. IMGT^®^ is specialized in the immunoglobulins (IG) or antibodies, T cell receptors (TR), major histocompatibility (MH), and IgSF and MhSF superfamilies. IMGT^®^ has been built on the IMGT-ONTOLOGY axioms and concepts, which bridged the gap between genes, sequences and three-dimensional (3D) structures. The concepts include the IMGT^®^ standardized keywords (identification), IMGT^®^ standardized labels (description), IMGT^®^ standardized nomenclature (classification), IMGT unique numbering and IMGT Colliers de Perles (numerotation). IMGT^®^ comprises seven databases, 15,000 pages of web resources and 17 tools. IMGT^®^ tools and databases provide a high-quality analysis of the IG from fish to humans, for basic, veterinary and medical research, and for antibody engineering and humanization. They include, as examples: IMGT/V-QUEST and IMGT/JunctionAnalysis for nucleotide sequence analysis and their high-throughput version IMGT/HighV-QUEST for next generation sequencing, IMGT/DomainGapAlign for amino acid sequence analysis of IG domains, IMGT/3Dstructure-DB for 3D structures, contact analysis and paratope/epitope interactions of IG/antigen complexes, and the IMGT/mAb-DB interface for therapeutic antibodies and fusion proteins for immunological applications (FPIA).

## 1. IMGT^®^: The Birth of Immunoinformatics

IMGT^®^, the international ImMunoGeneTics information system^®^ [1,2], was created in 1989 by Marie-Paule Lefranc at Montpellier, France (CNRS and Montpellier University). The founding of IMGT^®^ marked the advent of immunoinformatics, a new science, which emerged at the interface between immunogenetics and bioinformatics. For the first time, immunoglobulin (IG) or antibody and T cell receptor (TR) variable (V), diversity (D), joining (J) and constant (C) genes were officially recognized as “genes” as well as the conventional genes [3,4,5,6]. This major breakthrough allowed genes and data of the complex and highly diversified adaptive immune responses to be managed in genomic databases and tools.

The adaptive immune response was acquired by jawed vertebrates (or *gnathostomata*) more than 450 million years ago and is found in all extant jawed vertebrate species from fishes to humans. It is characterized by a remarkable immune specificity and memory, which are properties of the B and T cells owing to an extreme diversity of their antigen receptors. The specific antigen receptors comprise the IG or antibodies of the B cells and plasmacytes [3], and the TR [4]. The IG recognize antigens in their native (unprocessed) form, whereas the TR recognize processed antigens, which are presented as peptides by the highly polymorphic major histocompatibility (MH, in humans HLA for human leucocyte antigens) proteins.

The potential antigen receptor repertoire of each individual is estimated to comprise about 2 × 10^12^ different IG and TR, and the limiting factor is only the number of B and T cells that an organism is genetically programmed to produce [3,4]. This huge diversity results from the complex molecular synthesis of the IG and TR chains and, more particularly, of their variable domains (V-DOMAIN) which, at their N-terminal end, recognize and bind the antigens [3,4]. The IG and TR synthesis includes several unique mechanisms that occur at the DNA level: combinatorial rearrangements of the V, D and J genes that code the V-DOMAIN (the V-(D)-J being spliced to the C gene that encodes the C-REGION in the transcript); exonuclease trimming at the ends of the V, D and J genes; and random addition of nucleotides by the terminal deoxynucleotidyl transferase (TdT) that creates the junctional N-diversity regions, and later during B cell differentiation, for the IG, somatic hypermutations, gene conversion (e.g., in birds), and class or subclass switch in higher vertebrates [3,4].

**Figure 1:**
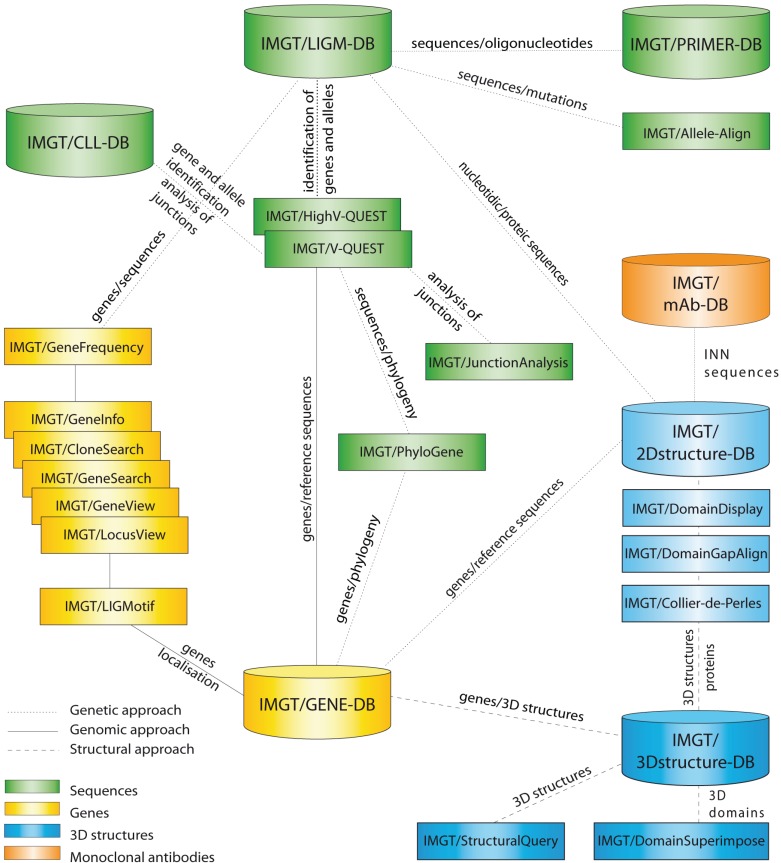
IMGT^®^, the international ImMunoGeneTics information system^®^ [1,2]. Databases are shown as cylinders and tools as rectangles. The web resources are not shown.

IMGT^®^ manages the diversity and complexity of the IG and TR and the polymorphism of the MH of humans and other vertebrates. IMGT^®^ is also specialized in the other proteins of the immunoglobulin superfamily (IgSF) and MH superfamily (MhSF) and related proteins of the immune system (RPI) of vertebrates and invertebrates [2]. IMGT^®^ provides a common access to standardized data from genome, proteome, genetics, and two-dimensional (2D) and three-dimensional (3D) structures. IMGT^®^ is the acknowledged high-quality integrated knowledge resource in immunogenetics for exploring immune functional genomics. IMGT^®^ comprises seven databases (for sequences, genes, and 3D structures) [7,8,9,10,11,12] and 17 online tools [13,14,15,16,17,18,19,20,21,22,23,24,25,26,27,28] (Figure 1), as well as more than 15,000 pages of web resources (e.g., IMGT Scientific chart, IMGT Repertoire, IMGT Education > Aide-mémoire [29], The IMGT Medical page, The IMGT Veterinary page, The IMGT Biotechnology page, The IMGT Immunoinformatics page) [2]. IMGT^®^ is the global reference in immunogenetics and immunoinformatics [30,31,32,33,34,35,36,37,38,39,40,41,42,43,44,45]. Its standards have been endorsed by the World Health Organization-International Union of Immunological Societies (WHO-IUIS) Nomenclature Committee since 1995 (first IMGT^®^ online access at the 9th International Congress of Immunology, San Francisco, CA, USA) [46,47] and the WHO International Nonproprietary Names (INN) Programme [48,49].

The accuracy and the consistency of the IMGT^®^ data are based on IMGT-ONTOLOGY [50,51,52], the first, and so far unique, ontology for immunogenetics and immunoinformatics [50,51,52,53,54,55,56,57,58,59,60,61,62,63,64,65,66,67,68,69]. IMGT-ONTOLOGY manages the immunogenetics knowledge through diverse facets that rely on seven axioms: IDENTIFICATION, DESCRIPTION, CLASSIFICATION, NUMEROTATION, LOCALIZATION, ORIENTATION, and OBTENTION [51,52,56]. The concepts generated from these axioms led to the elaboration of the IMGT^®^ standards that constitute the IMGT Scientific chart: e.g., IMGT^®^ standardized keywords (IDENTIFICATION) [57], IMGT^®^ standardized labels (DESCRIPTION) [58], IMGT^®^ standardized gene and allele nomenclature (CLASSIFICATION) [59], IMGT unique numbering [60,61,62,63,64,65] and its standardized graphical 2D representation or IMGT Colliers de Perles [66,67,68,69] (NUMEROTATION).

With a focus on IG, we first review the fundamental information generated from these IMGT-ONTOLOGY concepts which led to the IMGT Scientific chart rules. The major IMGT^®^ tools and databases used for IG repertoire analysis, antibody engineering and humanization, and IG/Ag structures are then briefly presented: IMGT/V-QUEST [13,14,15,16,17,18] for the analysis of rearranged nucleotide sequence with the results of the integrated IMGT/JunctionAnalysis [19,20]; IMGT/Automat [21,22] and IMGT/Collier-de-Perles tool [27]; IMGT/HighV-QUEST, the high-throughput version for Next-Generation Sequencing (NGS) [23,24]; IMGT/DomainGapAlign [10,25,26] for amino acid sequence analysis; IMGT/3Dstructure-DB for 3D structures [9,10,11]; and its extension, IMGT/2Dstructure-DB (for antibodies and other proteins for which the 3D structure is not available). IMGT^®^ tools and databases run against IMGT reference directories built from sequences annotated in IMGT/LIGM-DB [7], the IMGT^®^ nucleotide database (176,806 sequences from 346 species in October 2014), and from IMGT/GENE-DB [8], the IMGT^®^ gene database (3464 genes and 5118 alleles from 21 species, of which there were 710 genes and 1439 alleles for *Homo sapiens* and 868 genes and 1318 alleles for *Mus musculus* in October 2014).

An interface, IMGT/mAb-DB [12], has been developed to provide an easy access to therapeutic antibody amino acid sequences (links to IMGT/2Dstructure-DB) and structures (links to IMGT/3Dstructure-DB, if 3D structures are available). IMGT/mAb-DB data include monoclonal antibodies (mAb, INN suffix –mab) (a –mab is defined by the presence of at least an IG variable domain) and fusion proteins for immune applications (FPIA, INN suffix –cept) (a –cept is defined by a receptor fused to an Fc) from the WHO-INN programme [48,49]. This database also includes a few composite proteins for clinical applications (CPCA) (e.g., protein or peptide fused to an Fc for only increasing their half-life, identified by the INN prefix ef–) and some RPI used, unmodified, for clinical applications.

The unified IMGT^®^ approach is of major interest for bridging knowledge from IG repertoire in normal and pathological situations [70,71,72,73,74,75], IG allotypes and immunogenicity [76,77,78], NGS repertoire [23,24], antibody engineering and humanization [33,40,41,42,79,80,81,82,83,84,85,86].

## 2. Fundamental Information from IMGT-ONTOLOGY Concepts

### 2.1. IDENTIFICATION: IMGT^®^ Standardized Keywords

More than 325 IMGT^®^ standardized keywords (189 for sequences and 137 for 3D structures) were precisely defined [57]. They represent the controlled vocabulary assigned during the annotation process and allow standardized search criteria for querying the IMGT^®^ databases and for the extraction of sequences and 3D structures. They have been entered in BioPortal [87] at the National Center for Biomedical Ontology (NCBO) in 2010.

Standardized keywords are assigned at each step of the molecular synthesis of an IG. Those assigned to a nucleotide sequence are found in the “DE” (definition) and “KW” (keyword) lines of the IMGT/LIGM-DB files [7]. They characterize, for instance, the gene type, the configuration type and the functionality type [57]. There are six gene types: variable (V), diversity (D), joining (J), constant (C), conventional-with-leader, and conventional-without-leader. Four of them (V, D, J, and C) identify the IG and TR genes and are specific to immunogenetics. There are four configuration types: germline (for the V, D, and J genes before DNA rearrangement), rearranged (for the V, D, and J genes after DNA rearrangement), partially-rearranged (for D gene after only one DNA rearrangement) and undefined (for the C gene and for the conventional genes, which do not rearrange). The functionality type depends on the gene configuration. The functionality type of genes in germline or undefined configuration is functional (F), ORF (for “open reading frame”), or pseudogene (P). The functionality type of genes in rearranged or partially-rearranged configuration is either productive (no stop codon in the V-(D)-J region and in-frame junction) or unproductive (stop codon(s) in the V-(D)-J region, and/or out-of-frame junction).

The 20 usual amino acids (AA) have been classified in 11 IMGT physicochemical classes (IMGT^®^ [1], IMGT Education > Aide-mémoire > Amino acids). The amino acid changes are described according to the hydropathy (three classes), volume (five classes) and IMGT physicochemical classes (11 classes) [29]. For example Q1 > E (+ + −) means that in the amino acid change (Q > E), the two amino acids at codon 1 belong to the same hydropathy (+) and volume (+) classes but to different IMGT physicochemical properties (−) classes [29]. Four types of AA changes are identified in IMGT^®^: very similar (+ + +), similar (+ + −, + − +), dissimilar (− − +, − + −, + − −), and very dissimilar (− − −).

### 2.2. DESCRIPTION: IMGT^®^ Standardized Labels

**Figure 2:**
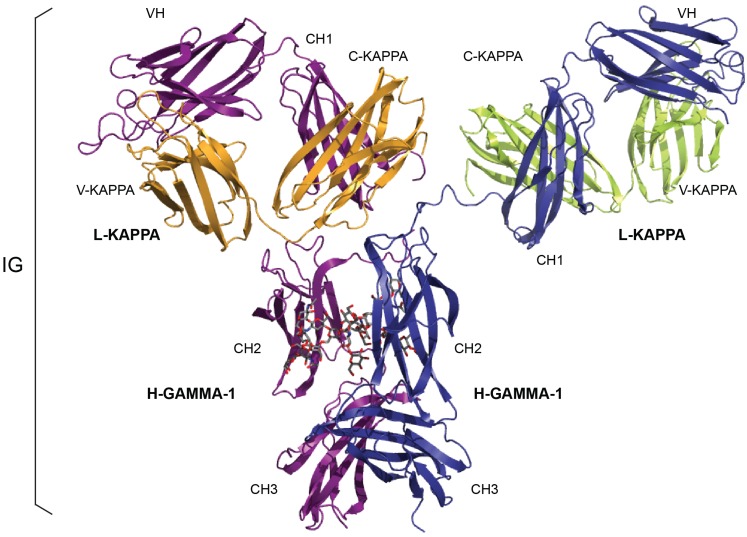
An immunoglobulin (IG) or antibody. *In vivo*, an IG or antibody is anchored in the membrane of a B cell as part of a signaling B cell receptor (BcR = membrane IG + CD79) or, as shown here, is secreted [3]. An IG is made of two identical heavy (H, for IG-HEAVY) chains and two identical light (L, for IG-LIGHT) chains [3]. An IG comprises 12 domains (for example, IgG1, shown here) or 14 domains (IgM or IgE). The V-DOMAIN of each chain (green online) and the C-DOMAIN, one for each L chain and three for each H chain (blue online) are highlighted. The light chain (here, L-KAPPA) is made of a variable domain (V-DOMAIN, here, V-KAPPA) at the N-terminal end and a constant domain (C-DOMAIN, here, C-KAPPA) at the C-terminal end. The heavy chain (here, H-GAMMA-1) is made of a VH (at the N-terminal end) and of three CH (four for H-MU or H-EPSILON) (Table 1) [3]. The structure is that of the antibody b12, an IgG1-kappa, and so far the only complete human IG crystallized (1hzh from IMGT/3Dstructure-DB [1]).

More than 560 IMGT^®^ standardized labels (277 for sequences and 285 for 3D structures) were precisely defined [58]. They are written in capital letters (no plural) to be recognizable without creating new terms. Standardized labels assigned to the description of sequences are found in the “FT” (feature) lines of the IMGT/LIGM-DB files [7]. Querying these labels represent a big plus compared to the generalist databases (GenBank/European Nucleotide Archive (ENA)/DNA Data Bank of Japan (DDBJ)). Thus it is possible to query for the “CDR3-IMGT’ of the human rearranged productive sequences of IG-Heavy-Gamma (e.g., 1733 CDR3-IMGT obtained, with their sequences at the nucleotide or amino acid level). The core labels include V-REGION, D-REGION, J-REGION, and C-REGION which correspond to the coding region of the V, D, J and C genes. IMGT structure labels for IG chains and domains are illustrated with the example of an human IgG1 (Figure 2). Correspondence between human IG structure labels and sequence labels are provided in Table 1. These labels are necessary for a standardized description of the IG sequences and structures in databases and tools [58].

Highly conserved amino acids at a given position in a domain have IMGT labels [58]. Thus three amino acid labels are common to the V and C domains: 1st-CYS (cysteine C at position 23), CONSERVED-TRP (tryptophan W at position 41), and 2nd-CYS (C at position 104) [60,61,62,63,65]. Two other labels are characteristics of the V-DOMAIN and correspond to the first amino acid of the canonical F/W-G-X-G motif (where F is phenylalanine, W tryptophan, G glycine, and X any amino acid) encoded by the J-REGION: J-PHE or J-TRP (F or W at position 118) [60,61,62,65].

**Table 1:** Immunoglobulin (IG) receptor, chain and domain structure labels and correspondence with sequence labels.

IG Structure Labels (IMGT/3Dstructure-DB)				Sequence Labels (IMGT/LIGM-DB)
Receptor ^a^	Chain ^b^	Domain Description Type	Domain ^c^	Region
IG-GAMMA-1_KAPPA	L-KAPPA	V-DOMAIN	V-KAPPA	V-J-REGION
		C-DOMAIN	C-KAPPA	C-REGION
	H-GAMMA-1	V-DOMAIN	VH	V-D-J-REGION
		C-DOMAIN	CH1	C-REGION ^d^
		C-DOMAIN	CH2	
		C-DOMAIN	CH3	
IG-MU_LAMBDA	L-LAMBDA	V-DOMAIN	V-LAMBDA	V-J-REGION
		C-DOMAIN	C-LAMBDA-1	C-REGION
	H-MU	V-DOMAIN	VH	V-D-J-REGION
		C-DOMAIN	CH1	C-REGION *^d^*
		C-DOMAIN	CH2	
		C-DOMAIN	CH3	
		C-DOMAIN	CH4 ^e^	

^a^ Labels are shown for two examples of IG (*Homo sapiens* IgG1-kappa and IgM-lambda). An IG (“Receptor”) (Figure 1) is made of two identical heavy (H, for IG-HEAVY) chains and two identical light (L, for IG-LIGHT) chains (“Chain‘) and usually comprises 12 (e.g., IgG1) or 14 (e.g., IgM) domains. Each chain has a N-terminal V-DOMAIN (or V-(D)-J-REGION, encoded by the rearranged V-(d)-J genes), whereas the remaining of the chain is the C-REGION (encoded by a C gene). The IG C-REGION comprises one C-DOMAIN (C-KAPPA or C-LAMBDA) for the L chain, or several C-DOMAIN (CH) for the H chain [3]. ^b^ The kappa (L-KAPPA) or lambda (L-LAMBDA) light chains may associate to any heavy chain isotype (e.g., H-GAMMA-1, H-MU). In humans, there are 9 isotypes, H-MU, H-DELTA, H-GAMMA-3, H-GAMMA-1, H-ALPHA1, H-GAMMA2, H-GAMMA-4, H-EPSILON, H-ALPHA2 (listed in the order 5'–3' in the IGH locus of the IGHC genes which encode the constant region of the heavy chains [3] (IMGT^®^ [1], IMGT Repertoire). ^c^ The IG V-DOMAIN includes VH (for the IG heavy chain) and VL (for the IG light chain). In higher vertebrates, the VL is V-KAPPA or V-LAMBDA, whereas in fishes, the VL is V-IOTA. The C-DOMAIN includes CH (for the IG heavy chain, the number of CH per chain depending on the isotype [3]) and CL (for the IG light chain). In higher vertebrates, the CL is C-KAPPA or C-LAMBDA, whereas in fishes, the CL is C-IOTA. ^d^ The heavy chain C-REGION also includes the HINGE-REGION for the H-ALPHA, H-DELTA and H-GAMMA chains and, for membrane IG (mIG), the CONNECTING-REGION (CO), TRANSMEMBRANE-REGION (TM) and CYTOPLASMIC-REGION (CY); for secreted IG (sIG), the C-REGION includes CHS instead of CO, TM and CY. ^e^ For H-MU and H-EPSILON.

### 2.3. CLASSIFICATION: IMGT^®^ Standardized Genes and Alleles

The IMGT-ONTOLOGY CLASSIFICATION axiom was the trigger of immunoinformatics’ birth [45]. Indeed the IMGT^®^ concepts of classification allowed us, for the first time, to classify the antigen receptor genes (IG and TR) for any locus (e.g., immunoglobulin heavy (IGH), T cell receptor alpha (TRA)), for any gene configuration (germline, undefined, or rearranged) and for any species (from fishes to humans). In higher vertebrates, there are three IG major loci (other loci correspond to chromosomal orphon sets, genes of which are orphons, not used in the IG chain synthesis). The IG major loci include the immunoglobulin heavy (IGH), and for the light chains, the immunoglobulin kappa (IGK) and the immunoglobulin lambda (IGL) in higher vertebrates and the immunoglobulin iota (IGI) in fishes (IMGT^®^ [1], IMGT Repertoire).

Since the creation of IMGT^®^ in 1989, at New Haven during the 10th Human Genome Mapping Workshop (HGM10), the standardized classification and nomenclature of the IG and TR of humans and other vertebrate species have been under the responsibility of the IMGT Nomenclature Committee (IMGT-NC).

IMGT^®^ gene and allele names are based on the concepts of classification of “Group”, “Subgroup”, “Gene” and “Allele” [59]. “Group” allows classification of a set of genes that belong to the same multigene family, within the same species or between different species. For example, there are 10 groups for the IG of higher vertebrates: IGHV, IGHD, IGHJ, IGHC, IGKV, IGKJ, IGKC, IGLV, IGLJ, and IGLC. “Subgroup” allows classification of a subset of genes that belong to the same group and that, in a given species, share at least 75% identity at the nucleotide level, e.g., *Homo sapiens* IGHV1 subgroup. Subgroups, genes, and alleles are always associated to a species name. An allele is a polymorphic variant of a gene, which is characterized by the mutations of its sequence at the nucleotide level, identified in its core sequence, and compared to the gene allele reference sequence, designated as allele *01. For example, *Homo sapiens* IGHV1-2*01 is the allele *01 of the *Homo sapiens* IGHV1-2 gene that belongs to the *Homo sapiens* IGHV1 subgroup which itself belongs to the IGHV group. For the IGH locus, the constant genes are designated by the letter (and eventually number) corresponding to the encoded isotypes (IGHM, IGHD, IGHG3…), instead of using the letter C. IG and TR genes and alleles are not italicized in publications. IMGT-ONTOLOGY concepts of classification have been entered in the NCBO BioPortal.

The IMGT^®^ IG and TR gene names [3,4,5,6] were approved by the Human Genome Organisation (HUGO) Nomenclature Committee (HGNC) in 1999 [88,89] and were endorsed by the WHO-IUIS Nomenclature Subcommittee for IG and TR [46,47]. The IMGT^®^ IG and TR gene names are the official international reference and, as such, have been entered in IMGT/GENE-DB [8], in the Genome Database (GDB) [90], in LocusLink at the National Center for Biotechnology Information (NCBI) USA [91], in Entrez Gene (NCBI) when this database (now designated as “Gene”) superseded LocusLink [92], in NCBI MapViewer, in Ensembl at the European Bioinformatics Institute (EBI) [93], and in the Vertebrate Genome Annotation (Vega) Browser [94] at the Wellcome Trust Sanger Institute (UK). HGNC, Gene NCBI, Ensembl, and Vega have direct links to IMGT/GENE-DB [8]. IMGT^®^ human IG and TR genes were also integrated in IMGT-ONTOLOGY on the NCBO BioPortal and, on the same site, in the HUGO ontology and in the National Cancer Institute (NCI) Metathesaurus. Amino acid sequences of human IG and TR constant genes (e.g., *Homo sapiens* IGHM, IGHG1, IGHG2) were provided to UniProt in 2008. Since 2007, IMGT^®^ IG gene and allele names have been used for the description of the therapeutic mAb and FPIA of the WHO-INN program [48,49].

### 2.4. NUMEROTATION: IMGT Unique Numbering and IMGT Collier de Perles

The IMGT-ONTOLOGY NUMEROTATION axiom is acknowledged as the “IMGT^®^ Rosetta stone” that has bridged the biological and computational spheres in bioinformatics [38]. The IMGT^®^ concepts of numerotation comprise the IMGT unique numbering [60,61,62,63,64,65] and its graphical 2D representation the IMGT Collier de Perles [66,67,68,69]. Developed for and by the “domain”, these concepts integrate sequences, structures, and interactions into a standardized domain-centric knowledge for functional genomics. The IMGT unique numbering has been defined for the variable V domain (V-DOMAIN of the IG and TR, and V-LIKE-DOMAIN of IgSF other than IG and TR) [60,61,62], the constant C domain (C-DOMAIN of the IG and TR, and C-LIKE-DOMAIN of IgSF other than IG and TR) [63] and the groove G domain (G-DOMAIN of the MH, and G-LIKE-DOMAIN of MhSF other than MH) [64]. Thus the IMGT unique numbering and IMGT Collier de Perles provide a definitive and universal system across species, including invertebrates, for the sequences and structures of the V, C and G domains of IG, TR, MH, IgSF and MhSF [65,69,84].

#### 2.4.1. IG V-DOMAIN

##### 2.4.1.1. V-DOMAIN Definition and Main Characteristics

The V-DOMAIN of the IG and TR are encoded by V-(D)-J rearrangements [3,4]. Thus, the VH of an IG heavy chain corresponds to a V-D-J-REGION, whereas the V-KAPPA or V-LAMBDA (or V-IOTA for fishes) of an IG light chain corresponds to a V-J-REGION (Table 1).

**Figure 3:**
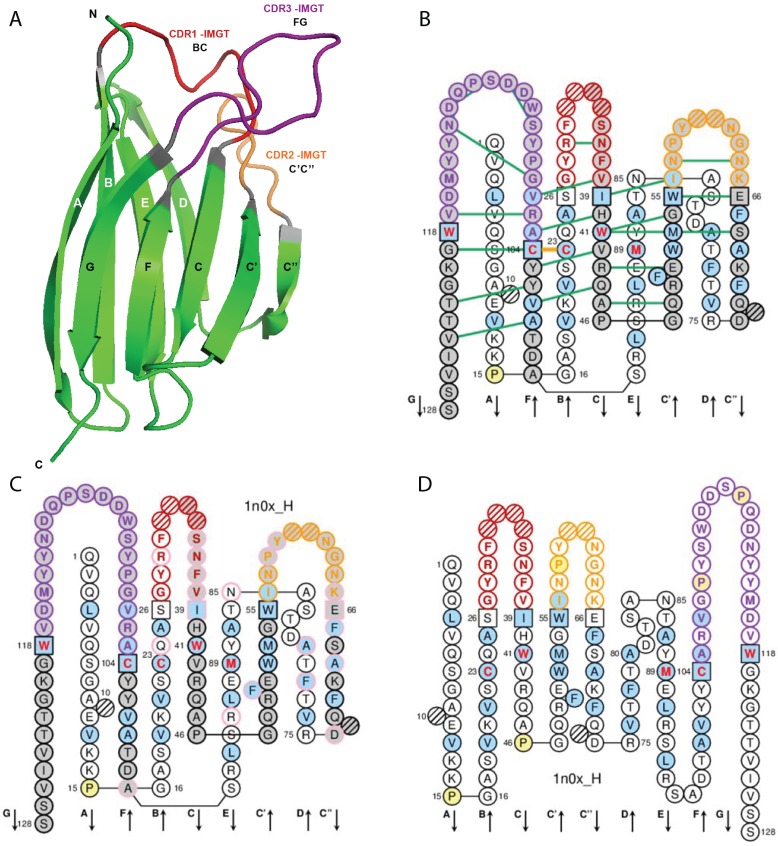
Variable (V) domain. An IG VH (V-DOMAIN) is shown as example. (**A**) 3D structure ribbon representation with the IMGT strand and loop delimitations [62]; (**B**) IMGT Collier de Perles on two layers with hydrogen bonds. The IMGT Collier de Perles on two layers show, in the forefront, the GFCC'C'' strands (forming the sheet located at the interface VH/VL of the IG) and, in the back, the ABED strands. The IMGT Collier de Perles with hydrogen bonds (green lines online, only shown here for the GFCC'C'' sheet) is generated by the IMGT/Collier-de-Perles tool integrated in IMGT/3Dstructure-DB, from experimental 3D structure data [9,10,11]; (**C**) IMGT Collier de Perles on two layers generated from IMGT/DomainGapAlign [10,25,26]. Pink circles (online) indicate amino acid changes compared to the closest genes and alleles from the IMGT reference directory; (**D**) IMGT Collier de Perles on one layer. Amino acids are shown in the one-letter abbreviation All proline (P) are shown online in yellow. IMGT anchors are in square. Hatched circles are IMGT gaps according to the IMGT unique numbering for V domain [62,65]. Positions with bold (online red) letters indicate the four conserved positions that are common to a V domain and to a C domain: 23 (1st-CYS), 41 (CONSERVED-TRP), 89 (hydrophobic), 104 2nd-CYS) [60,61,62,63,65], and the fifth conserved position, 118 (J-TRP or J-PHE) which is specific to a V-DOMAIN and belongs to the motif F/W-G-X-G that characterizes the J-REGION [62,65] (Table 2). The hydrophobic amino acids (hydropathy index with positive value: I, V, L, F, C, M, A) and tryptophan (W) [29] found at a given position in more than 50% of sequences are shown (online with a blue background color). Arrows indicate the direction of the beta strands and their designations in 3D structures. IMGT color menu for the CDR-IMGT of a V-DOMAIN indicates the type of rearrangement, V-D-J (for a VH, red, orange and purple) or V-J (for V-KAPPA or V-LAMBDA, blue, green and greenblue) [3]. The identifier of the chain to which the VH domain belongs is 1n0x_H (from the *Homo sapiens* b12 Fab) in IMGT/3Dstructure-DB [1]. The CDR-IMGT lengths of this VH are [8.8.20] and the FR-IMGT are [25.17.38.11]. The 3D ribbon representation was obtained using PyMOL [97] and “IMGT numbering comparison” of 1n0x_H (VH) from IMGT/3Dstructure-DB [1].

A V-DOMAIN (Figure 3) comprises about 100 amino acids and is made of nine antiparallel beta strands (A, B, C, C', C'', D, E, F, and G) linked by beta turns (AB, CC', C''D, DE, and EF) and three loops (BC, C'C'', and FG), forming a sandwich of two sheets [ABED] [GFCC'C''] [60,61,62,65]. The sheets are closely packed against each other through hydrophobic interactions, giving a hydrophobic core, and joined together by a disulfide bridge between a first highly conserved cysteine (1st-CYS) in the B strand (in the first sheet) and a second, equally conserved cysteine (2nd-CYS) in the F strand (in the second sheet) [60,61,62,65].

##### 2.4.1.2. V-DOMAIN Strands and Loops (FR-IMGT and CDR-IMGT)

The V-DOMAIN strands and loops and their delimitations and lengths, based on the IMGT unique numbering for V domain [60,61,62,65], are shown in Table 2. In the IG and TR V-DOMAIN, the three hypervariable loops BC, C'C'', and FG involved in the ligand recognition (native antigen for IG and pMH for TR) are designated complementarity determining regions (CDR-IMGT), whereas the strands form the framework region (FR-IMGT), which includes FR1-IMGT, FR2-IMGT, FR3-IMGT, and FR4-IMGT (Table 2). In the IMGT^®^ definitive system [84], the CDR-IMGT have accurate and unambiguous delimitations, in contrast to the CDR described in the literature. Correspondences between the IMGT unique numbering with other numberings, e.g., Kabat [95] or Chothia [96], are available in the IMGT Scientific chart. The correspondences with these previous and heterogenous numberings are useful for the interpretation of previously published data, but nowadays the usage of these numberings has become obsolete in regard of the development of immunoinformatics based on the IMGT^®^ standards [45,60,61,62,63,64,65,66,67,68,69,70,84] (IMGT^®^ [1], IMGT Scientific chart > Numbering > Correspondence between V numberings).

For a V-DOMAIN, the CDR1-IMGT or BC loop encompasses positions 27 to 38, the CDR2-IMGT or C'C'' loop, positions 56 to 65, and the CDR3-IMGT or FG loop, positions 105 to 117. The CDR3-IMGT encompasses the V-(D)-J junction that results from a V-J or V-D-J rearrangement [3,4] and is more variable in sequence and length than the CDR1-IMGT and CDR2-IMGT that are encoded by the V gene region only. For CDR3-IMGT of length > 13 AA, additional IMGT positions are added at the top of the loop between 111 and 112 (Table 3).

**Table 2:** IG V-DOMAIN strands and loops, IMGT positions and lengths, based on the IMGT unique numbering for V domain.

V-DOMAIN Strands and Loops ^a^	IMGT Position ^b^	Lengths ^c^	Characteristic IMGT Residue@ Position ^d^	V-DOMAIN FR-IMGT and CDR-IMGT
A-STRAND	1–15	15 (14 if gap at 10)		FR1-IMGT
B-STRAND	16–26	11	1st-CYS 23	
BC-LOOP	27–38	12 (or less)		CDR1-IMGT
C-STRAND	39–46	8	CONSERVED-TRP 41	FR2-IMGT
C’-STRAND	47–55	9		
C’C”-LOOP	56–65	10 (or less)		CDR2-IMGT
C”-STRAND	66–74	9 (or 8 if gap at 73)		FR3-IMGT
D-STRAND	75–84	10 (or 8 if gaps at 81, 82)		
E-STRAND	85–96	12	hydrophobic 89	
F-STRAND	97–104	8	2nd-CYS 104	
FG-LOOP	105–117	13 (or less, or more)		CDR3-IMGT
G-STRAND	118–128	11 (or 10)	V-DOMAIN J-PHE 118 or J-TRP 118 ^e^	FR4-IMGT

^a^ IMGT^®^ labels (concepts of description) are written in capital letters (no plural) [58]. Beta turns (AB, CC', C''D, DE or EF) are individualized only if they have additional amino acids compared to the standard description. If not, they are included in the strands. ^b^ based on the IMGT unique numbering for V domain (V-DOMAIN and V-LIKE-DOMAIN) [60,61,62,65]. ^c^ in number of amino acids (or codons). ^d^ IMGT Residue@Position is a given residue (usually an amino acid) or a given conserved property amino acid class, at a given position in a domain, based on the IMGT unique numbering [65]. ^e^ In the IG (and also) TR V-DOMAIN, the G-STRAND (or FR4-IMGT) is the C-terminal part of the J-REGION, with J-PHE or J-TRP 118 and the canonical motif F/W-G-X-G at positions 118–121 [3,4]. The JUNCTION refers to the CDR3-IMGT plus the two anchors 2nd-CYS 104 and J-PHE or J-TRP 118 [61,62]. The JUNCTION (positions 104–118) is therefore two amino acids longer than the corresponding CDR3-IMGT (positions 105–117) [61,62].

**Table 3:** IMGT additional positions for CDR3-IMGT.

CDR3-IMGT Lengths	IMGT Additional Positions for CDR3-IMGT Length > 13 AA ^a^									
---										
21	111	111.1	111.2	111.3	111.4	112.4	112.3	112.2	112.1	112
20	111	111.1	111.2	111.3	-	112.4	112.3	112.2	112.1	112
19	111	111.1	111.2	111.3	-	-	112.3	112.2	112.1	112
18	111	111.1	111.2	-	-	-	112.3	112.2	112.1	112
17	111	111.1	111.2	-	-	-	-	112.2	112.1	112
16	111	111.1	-	-	-	-	-	112.2	112.1	112
15	111	111.1	-	-	-	-	-	-	112.1	112
14	111	-	-	-	-	-	-	-	112.1	112

^a^ For CDR3-IMGT length > 13 AA, IMGT additional positions are created between positions 111 and 112 at the top of the CDR3-IMGT loop in the following order 112.1, 111.1, 112.2, 111.2, 112.3, 111.3, *etc.* For CDR3-IMGT length < 13 AA, IMGT gaps are created classically from the top of the loop, in the following order 111, 112, 110, 113, 109, 114, *etc.* (IMGT^®^ [1], IMGT Scientific chart > Numbering).

##### 2.4.1.3. IMGT Colliers de Perles

The V-DOMAIN strands and loops (FR-IMGT and CDR-IMGT) are visualized in the IMGT Colliers de Perles [66,67,68,69] which can be displayed on one layer (closer to the amino acid sequence) or on two layers (closer to the 3D structure) (Figure 3). The three loops, BC, C'C'', and FG (CDR1-IMGT, CDR2-IMGT, and CDR3-IMGT) are delimited by the IMGT anchors, which are shown in squares in the IMGT Colliers de Perles. IMGT anchors are positions that belong to strands and represent anchors for the loops of the V domains. IMGT anchors are the key and original concept of IMGT^®^ that definitively solved the ambiguous situation of different CDR lengths and delimitations found in the literature. The six anchors of a V-DOMAIN are positions 26 and 39 (anchors of the BC loop or CDR1-IMGT), 55 and 66 (anchors of the C'-C'' loop or CDR2-IMGT), 104 and 118 (anchors of the FG loop or CDR3-IMGT). The CDR3-IMGT anchors are highly conserved; they are C104 (2nd-CYS, in F strand) and F118 or W118 (J-PHE or J-TRP, in G strand). The JUNCTION of an IG or TR V-DOMAIN includes the anchors 104 and 118 and is therefore two amino acids longer than the corresponding CDR3-IMGT (positions 105–117).

In biological data, the lengths of the loops and strands are given by the number of occupied positions (unoccupied positions or “IMGT gaps” are represented with hatches in the IMGT Colliers de Perles (Figure 3) or by dots in alignments). The CDR-IMGT lengths are given in number of amino acids (or codons), into brackets and separated by dots: for example [9.6.9] means that the BC, C'C'' and FG loops (or CDR1-IMGT, CDR2-IMGT and CDR3-IMGT, respectively) have a length of 9, 6 and 9 amino acids (or codons), respectively. Similarly [25.17.38.11] means that the FR1-IMGT, FR2-IMGT, FR3-IMGT and FR4-IMGT have a length of 25, 17, 38 and 11 amino acids (or codons), respectively. Together, the four FR of a VH domain usually comprise 91 amino acids and the individual FR-IMGT lengths are [25.17.38.11], whereas the four FR of a VL domain usually comprise 89 amino acids and the individual FR-IMGT lengths are [26.17.36.10].

##### 2.4.1.4. Conserved Amino Acids

A V-DOMAIN has five characteristic amino acids at given positions (positions with bold (online red) letters in the IMGT Colliers de Perles). Four of them are highly conserved and hydrophobic [29] and are common to the C-DOMAIN: 23 (1st-CYS), 41 (CONSERVED-TRP), 89 (hydrophobic), and 104 (2nd-CYS). These amino acids contribute to the two major features shared by the V and C domain: the disulfide bridge (between the two cysteines 23 and 104) and the internal hydrophobic core of the domain (with the side chains of tryptophan W41 and amino acid 89). The fifth position, 118, is an anchor of the FG loop. It is occupied, in the V domains of IgSF other than IG or TR, by amino acids with diverse physicochemical properties [29]. In contrast, in IG and TR V-DOMAIN, the position 118 is occupied by remarkably conserved amino acids, which consist of a phenylalanine or a tryptophan encoded by the J-REGION and therefore designated J-TRP or J-PHE 118. The bulky aromatic side chains of J-TRP and J-PHE are internally orientated and structurally contribute to the V-DOMAIN hydrophobic core [62].

##### 2.4.1.5. Genomic Delimitation

A criterion used in the IMGT^®^ definitive system for the characterization of a V domain is its delimitation taking into account the exon delimitations, whenever appropriate [84]. The exon rule is not used for the delimitation of the 5' end of the first N-terminal domain of proteins with a leader (this includes the V-DOMAIN of the IG and TR chains). In those cases, the 5' end of the first N-terminal domain corresponds to the proteolytic site between the leader (L-REGION) and the coding region of the mature chain. The IG and TR V-DOMAIN is therefore delimited in 5' by a proteolytic site and in 3' at the genomic level by the splicing site of the J-REGION [58]. This IMGT^®^ genomic approach integrates the strands A and G, in contrast to structural alignments that usually lack these strands due to their poor structural conservation, and thus bridges the gap between genomic data (exon) and 3D structure (domain) [84].

#### 2.4.2. IG C-DOMAIN

##### 2.4.2.1. C-DOMAIN Definition and Main Characteristics

**Figure 4:**
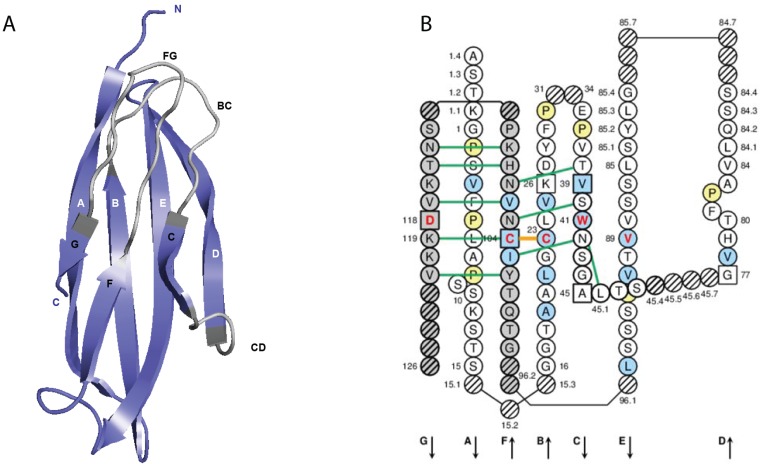

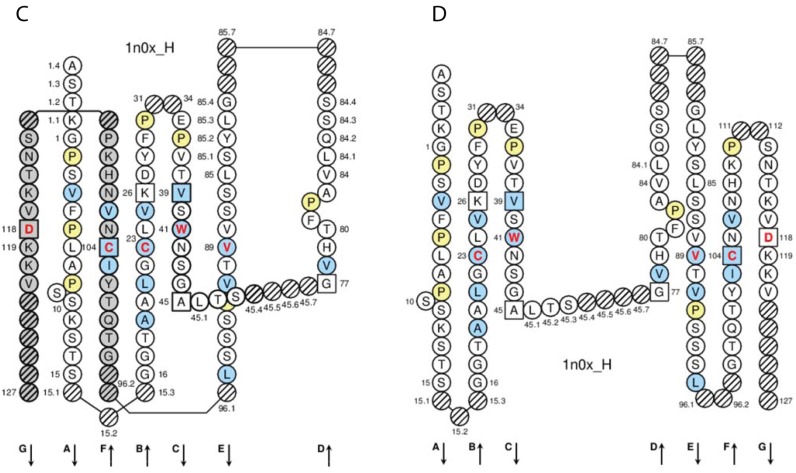
Constant (C) domain. An IG CH (C-DOMAIN) is shown as example. (**A**) 3D structure ribbon representation with the IMGT strand and loop delimitations [63]; (**B**) IMGT Collier de Perles on two layers with hydrogen bonds. The IMGT Colliers de Perles on two layers show, in the forefront, the GFC strands and, in the back, the ABED strands (located at the interface CH1/CL of the IG), linked by the CD transversal strand. The IMGT Collier de Perles with hydrogen bonds (green lines online, only shown here for the GFC sheet) is generated by the IMGT/Collier-de-Perles tool integrated in IMGT/3Dstructure-DB, from experimental 3D structure data [9,10,11]; (**C**) IMGT Collier de Perles on two layers from IMGT/DomainGapAlign [10,25,26]; (**D**) IMGT Colliers de Perles on one layer. Amino acids are shown in the one-letter abbreviation. All proline (P) are shown online in yellow. IMGT anchors are in square. Hatched circles are IMGT gaps according to the IMGT unique numbering for C domain [63,65]. Positions with bold (online red) letters indicate the four conserved positions that are common to a V domain and to a C domain: 23 (1st-CYS), 41 (CONSERVED-TRP), 89 (hydrophobic), 104 (2nd-CYS) [60,61,62,63,65] (Table 3), and position 118 which is only conserved in V-DOMAIN. The identifier of the chain to which the CH domain belongs is 1n0x_H (from the *Homo sapiens* b12 Fab, in IMGT/3Dstructure-DB, [1]). The 3D ribbon representation was obtained using PyMOL and “IMGT numbering comparison” of 1n0x_H (CH1) from IMGT/3Dstructure-DB [1].

A C-DOMAIN (Figure 4) comprises about 90–100 amino acids and is made of seven antiparallel beta strands (A, B, C, D, E, F, and G), linked by beta turns (AB, DE, and EF), a transversal strand (CD) and two loops (BC and FG), and forming a sandwich of two sheets [ABED] [GFC] [63,65]. A C-DOMAIN has a topology and a 3D structure similar to those of a V-DOMAIN but without the C' and C'' strands and the C'C'' loop, which is replaced by a transversal CD strand [63].

##### 2.4.2.2. C-DOMAIN Strands and Loops

The C-DOMAIN strands, turns and loops and their delimitations and lengths, based on the IMGT unique numbering for C domain [63,65], are shown in Table 4. Correspondences between the IMGT unique numbering with other numberings (Eu, Kabat) are available in the IMGT Scientific chart. The correspondences with these previous numberings are useful for the interpretation of previously published data but, as for the V domain, the usage of these previous numberings has become obsolete in regard to the development of immunoinformatics based on the IMGT^®^ standards [45,60,61,62,63,64,65,66,67,68,69,70,84] (IMGT^®^ [1], IMGT Scientific chart > Numbering > Correspondence between C numberings).

##### 2.4.2.3. IMGT Colliers de Perles

The C-DOMAIN lengths of the strands and loops are visualized in the IMGT Colliers de Perles [67,68,69], on one layer and two layers (Figure 4). There are six IMGT anchors in a C domain (four of them identical to those of a V domain): positions 26 and 39 (anchors of the BC loop), 45 and 77 (by extension, anchors of the CD strand as there is no C'-C'' loop in a C domain [63]), and 104 and 118 (anchors of the FG loop).

**Table 4:** IG C-DOMAIN strands, turns and loops, IMGT positions and lengths, based on the IMGT unique numbering for C domain.

C domain Strands, Turns and Loops ^a^	IMGT Position ^b^	Lengths ^c^	Characteristic IMGT Residue@Position ^d^
A-STRAND	1–15	15 (14 if gap at 10)	
AB-TURN	15.1–15.3	0–3	
B-STRAND	16–26	11	1st-CYS 23
BC-LOOP	27–31	10 (or less)	
	34–38		
C-STRAND	39–45	7	CONSERVED-TRP 41
CD-STRAND	45.1–45.9	0–9	
D-STRAND	77–84	8 (or 7 if gap at 82)	
DE-TURN	84.1–84.7	0–14	
	85.1–85.7		
E-STRAND	85–96	12	hydrophobic 89
EF-TURN	96.1–96.2	0–2	
F-STRAND	97–104	8	2nd-CYS 104
FG-LOOP	105–117	13 (or less, or more)	
G-STRAND	118–128	11 (or less)	

^a^ IMGT^®^ labels (concepts of description) are written in capital letters (no plural) [58]. ^b^ based on the IMGT unique numbering for C domain (C-DOMAIN and C-LIKE-DOMAIN) [63,65]. ^c^ in number of amino acids (or codons). ^d^ IMGT Residue@Position is a given residue (usually an amino acid) or a given conserved property amino acid class, at a given position in a domain, based on the IMGT unique numbering [65].

##### 2.4.2.4. Conserved Amino Acids

A C-DOMAIN has five characteristic amino acids at given positions (positions with bold (online red) letters in the IMGT Colliers de Perles). Four of them are highly conserved and hydrophobic [29] and are common to the V domain: 23 (1st-CYS), 41 (CONSERVED-TRP), 89 (hydrophobic) and 104 (2nd-CYS). As mentioned above, these amino acids contribute to the two major features shared by the V and C domain: the disulfide bridge (between the two cysteines 23 and 104) and the internal hydrophobic core of the domain (with the side chains of tryptophan W41 and amino acid 89). The fifth position, 118, is diverse and is characterized as being an FG loop anchor.

##### 2.4.2.5. Genomic Delimitation

The IG C-DOMAIN are delimited taking into account the exon delimitation, whenever appropriate [84]. As for the V-DOMAIN, this IMGT^®^ genomic approach integrates the strands A and G, which are absent of structural alignments [84].

#### 2.4.3. IMGT/Collier-de-Perles Tool

The IMGT/Collier-de-Perles tool [27], on the IMGT*^®^* Web site [1], is a generic tool which allows the users to draw IMGT Colliers de Perles [66,67,68,69] starting from their own domain amino acid sequences (sequences already gapped according to the IMGT unique numbering, using for example, IMGT/DomainGapAlign [10,25,26] (Table 5). IMGT Collier de Perles can be obtained for V and C domains (on one or two layers). The IMGT/Collier-de-Perles tool online can be customized to display the IG and TR CDR-IMGT according to the IMGT color menu and the amino acids according to their hydropathy or volume, or to the 11 IMGT physicochemical classes [29].

IMGT color menu for the CDR-IMGT of a V-DOMAIN indicates the type of rearrangement V-J or V-D-J [3,4]. Thus, the IMGT color menu for CDR1-IMGT, CDR2-IMGT and CDR3-IMGT is red, orange and purple for the IG VH (encoded by a V-D-J-REGION resulting from a V-D-J rearrangement), and blue, green and greenblue for the IG V-KAPPA or V-LAMBDA (encoded by a V-J-REGION resulting from a V-J rearrangement).

The IMGT/Collier-de-Perles tool is integrated in IMGT/DomainGapAlign [10,25,26] (analysis of user IG amino acid sequences containing V and/or C domains) and in IMGT/V-QUEST [13,14,15,16,17,18] (analysis of user nucleotide sequences containing V domains) (Table 5). IMGT Colliers de Perles for V and C domains are provided in IMGT/2Dstructure-DB (for amino acid sequences in the database) and in IMGT/3Dstructure-DB (on two layers with hydrogen bonds for the V or C domains, for 3D structures in the database) [9,10,11] (Table 5).

**Table 5:** IMGT^®^ tools and databases for the analysis of the IG V-DOMAIN and C-DOMAIN [1].

IMGT^®^ Tools	Results for IG V or C Domains (Nucleotide or Amino Acid Sequences)	Entry Types, Protocol References and Examples of Applications
IMGT/Collier-de-Perles [27]	Graphical 2D representation of IMGT Colliers de Perles [66,67,68,69]	User “IMGT gapped” V or C domain amino acid sequences (1 sequence per representation) [27]
IG V-DOMAIN nucleotide sequence and repertoire analysis		
IMGT/V-QUEST [13,14,15,16,17,18]	Introduction of IMGT gaps Identification of the closest V, D and J genes and alleles IMGT/Junction Analysis results [19,20] Description of mutations and amino acid changes Identification of indels and their correction [17] (option) IMGT/Automat annotation [21,22] IMGT Colliers de Perles [27]	- User nucleotide sequences of IG V-DOMAIN (1 to 50 sequences per analysis, and 1 to 10 sequences with the option “Search for insertions and deletions”) [17] - ***Applications***: somatic mutations in chronic lymphocytic leukemia (CLL) prognostic
IMGT/HighV-QUEST [22,23,24]	Introduction of IMGT gaps Identification of indels and their correction [17] (by default). Identification of the closest V, D and J genes and alleles IMGT/Junction Analysis results [19,20] Description of mutations and amino acid changes IMGT/Automat annotation [21,22] Statistical analysis [23] Characterization of the IMGT clonotypes (AA) [24]	- User NGS nucleotide sequences of V-DOMAIN (up to 500,000 sequences per run) ^a^ [23,24] - ***Applications***: IG immune repertoires and clonotypes in NGS
IG V-DOMAIN or C-DOMAIN amino acid sequence analysis		
IMGT/DomainGapAlign [10,25,26]	Introduction of IMGT gaps Identification of the closest genes and alleles Delimitation of the domains Description of amino acid (AA) changes IMGT Colliers de Perles [66,67,68,69] with highlighted AA changes (pink circles online)	- User amino acid sequences of V-DOMAIN or C-DOMAIN (1 to several sequences of same domain type) [25,26] - ***Applications***: IMGT antibody engineering and humanization for V and C
IMGT^®^ databases	Results for IG V-DOMAIN or C-DOMAIN (amino acid sequences or structures)	Number of entries and examples of applications
IMGT/3Dstructure-DB [9,10,11]	Identification of the closest genes and alleles IMGT/DomainGapAlign results [10,25,26] IMGT Collier de Perles [66,67,68,69] (on two layers with hydrogen bonds for V and C) Contact analysis between a pair of domains or between a domain and a ligand Renumbered IMGT files IMGT numbering comparison	- 2071 IG (including1327 IG/Ag complexes) on a total of 3153 entries ^b^ - ***Applications***: identification of the paratope and epitope in IG/AG complexes
IMGT/2Dstructure-DB [11] *	Identification of the closest genes and alleles IMGT/DomainGapAlign results [10,25,26] IMGT Collier de Perles [66,67,68,69] Renumbered IMGT files	- 548 IG (212 INN and 336 Kabat) on a total of 561 amino acid sequence entries ^b^ * - ***Applications***: from gene to structures in the absence of 3D

An asterisk (*) indicates that parts of the protocol dealing with 3D structures (hydrogen bonds in IMGT Colliers de Perles on two layers, Contact analysis) are not relevant, otherwise all other queries and results are similar to IMGT/3Dstructure-DB. ^a^ in October 2014, more than 4.38 billions of sequences analyzed by IMGT/HighV-QUEST, by 973 users from 40 countries (45% users from USA, 36% from EU, 19% from the remaining world). ^b^ in October 2014.

## 3. IMGT^®^ Tools for IG V-DOMAIN and C-DOMAIN Analysis

### 3.1. IMGT/V-QUEST

#### 3.1.1. IMGT/V-QUEST Tool

IMGT/V-QUEST [13,14,15,16,17,18] is the IMGT^®^ online tool for the analysis of nucleotide sequences of the IG and TR V-DOMAIN (Table 5). IMGT/V-QUEST identifies the variable (V), diversity (D), and junction (J) genes in rearranged IG and TR sequences and, for the IG, the nucleotide (nt) mutations and amino acid (AA) changes resulting from somatic hypermutations by comparison with the IMGT/V-QUEST reference directory. The tool integrates IMGT/JunctionAnalysis [19,20] for the detailed characterization of the V-D-J or V-J junctions, IMGT/Automat [21,22] for a complete sequence annotation, and IMGT/Collier-de-Perles [27].

The IMGT/V-QUEST most important functionalities include: introduction of “IMGT gaps” in the user nucleotide sequences (and in its translation); alignments and identification of the genes and alleles with the closest germline V, D, and J genes; analysis of the junctions; analysis of somatic hypermutations and amino acid changes; and, if the option “Search for insertions and deletions” was selected, identification of insertions and deletions (indels) and their correction. Customized parameters and results provided by IMGT/V-QUEST and IMGT/JunctionAnalysis have been described elsewhere [13,14,15,16,17,18,70].

#### 3.1.2. IMGT/V-QUEST Reference Directory

The IMGT/V-QUEST reference directory [2] comprises the IMGT reference sequences (nt), against which IMGT/V-QUEST is running. It includes the germline V, D and J nucleotide sequences of the core regions (V-REGION, D-REGION, and J-REGION) from all functional (F) genes and alleles, all open reading frame (ORF) and all in-frame pseudogenes (P) alleles from IMGT/GENE-DB [8]. It is organized per species and per locus in different sets (“F+ORF+ in-frame P”, being displayed by default online).

By definition, the IMGT/V-QUEST reference directory contains one reference sequence for each allele. By default, the user sequences are compared with all alleles of the genes of the selected set. However, the option “With allele *01 only” is useful for: (1) “Detailed view”, if the user sequences need to be compared with different genes; and (2) “Synthesis view”, if the user sequences that use the same gene need to be aligned together (independently of the allelic polymorphism) [15,17,18].

The IMGT/V-QUEST reference directories have been set up for species which have been extensively studied, such as human and mouse. This also holds for the other species or taxons with incomplete IMGT reference directory sets. In those cases, results should be interpreted considering the status of the IMGT reference directory (information on the updates on the IMGT^®^ Web site). Links to the IMGT/V-QUEST reference directory sets are available from the IMGT/V-QUEST Welcome page [15,17,18].

### 3.2. IMGT/HighV-QUEST

#### 3.2.1. IMGT/HighV-QUEST for NGS Repertoire Analysis

IMGT/HighV-QUEST [23], created in October 2010, is the high-throughput version of IMGT/V-QUEST. It is so far the only online tool available on the Web for the direct analysis of complete IG and TR V-DOMAIN nt sequences from NGS. It analyzes V-DOMAIN nt sequences, without the need of computational read assembly [23,24]. IMGT/HighV-QUEST analyzes up to 500,000 sequences per run and performs statistical analysis on the results [23,24] (Table 5), with the same degree of resolution and high-quality results as IMGT/V-QUEST [13,14,15,16,17,18,70]. IMGT/HighV-QUEST represents a major breakthrough for the analysis and the comparison of the antigen receptor V-DOMAIN repertoires and immunoprofilings of the adaptive immune response [23,24,70].

The functionalities of IMGT/HighV-QUEST include: the introduction of IMGT gaps; the identification of indels and their correction [17] (by default); the identification of the closest V, D, and J genes and alleles; the IMGT/JunctionAnalysis results; the description of mutations and amino acid changes; the annotation by IMGT/Automat; the NGS statistical analysis; and the characterization of the IMGT clonotypes (AA) [23,24,70] (Table 5)*.*

As for the other IMGT^®^ databases and tools, IMGT/HighV-QUEST is freely available for academics. However, the IMGT/HighV-QUEST Welcome page requires user identification and provides, for new users, a link to register. User identification has been set to avoid nonrelevant use and overload of the server, and to contact the user if needed. The user identification gives access to the IMGT/HighV-QUEST Search page.

#### 3.2.2. IMGT/HighV-QUEST for IMGT^®^ Clonotype (AA) Analysis

##### 3.2.2.1. IMGT Clonotypes (AA) Diversity

In the literature, clonotypes are defined differently, depending on the experiment design (functional specificity) or available data. Thus, a clonotype may denote either a complete antigen receptor (e.g., IgG1-kappa), or only one of the two chains of the receptor (e.g., H or L), or one domain (e.g., VH), or the CDR3 sequence of a domain. Moreover the sequence can be at the amino acid (AA) or nucleotide (nt) level, and this is rarely specified. Therefore, the goal of IMGT^®^ was first of all to define clonotypes and their properties, which could be identified and characterized by IMGT/HighV-QUEST, unambiguously [24].

In IMGT^®^, the clonotype, designated as “IMGT clonotype (AA)”, is defined by a unique V-(D)-J rearrangement (with IMGT gene and allele names determined by IMGT/HighV-QUEST at the nucleotide (nt) level) and a unique CDR3-IMGT AA (in-frame) junction sequence [24]. For identifying “IMGT clonotypes (AA)” in a given IMGT/HighV-QUEST dataset, the “1 copy” are filtered to select for sequences with in-frame junction, conserved anchors 104 and 118 (“C” is 2nd-CYS 104, and “F” or “W’ is the J-PHE or J-TRP 118) and for V and J functional or ORF, and “single allele” (for V and J) [24].

By essence, an “IMGT clonotype (AA)” is “unique” for a given dataset. For that reason, each “IMGT clonotype (AA)”, in a given dataset, has a unique set identifier and, importantly, has a unique representative sequence selected by IMGT/HighV-QUEST among the “1 copy” “single allele” (for V and J), based on the highest percent of identity of the V-REGION (“V %”) compared to that of the closest germline, and/or on the sequence length (thus the most complete V-REGION) [24,70].

##### 3.2.2.2. IMGT Clonotypes (AA) Expression

Clonotype expression is the number of sequences that can be assigned to each IMGT clonotype (AA). The total number of sequences assigned to each given “IMGT clonotype (AA)” is calculated by a stepwise procedure that aggregates sequences to the “IMGT clonotype (AA)”, and insures that high-quality and specific characterization of the “IMGT clonotype (AA)” remains unaltered) [24,70].

##### 3.2.2.3. IMGT^®^ Standardized Diversity and Expression Immunoprofiles

For the first time for NGS antigen receptor data analysis, the IMGT^®^ standardized approach allows a clear distinction and accurate evaluation between the clonal diversity (nb of “IMGT clonotypes (AA)”), and the clonal expression (total nb of sequences assigned, unambiguously, to a given “IMGT clonotype (AA)”) [24,70]. These assignments are clearly described and visualized in detail so the user always has the means of checking clonotypes individually. Indeed, the sequences of each “1 copy” assigned to a given “IMGT clonotype (AA)” are available in “Sequences file” [24,70]. The user can easily perform an analysis of these sequences online with IMGT/V-QUEST (up to 10 sequences, selecting “Synthesis view display” and the option “Search for insertions and deletions”), and/or with IMGT/JunctionAnalysis (up to 5000 junction sequences), which provide a visual representation familiar to the IMGT^®^ users.

Clonal diversity is also visualized in the online results with histograms which represent the number of IMGT clonotypes (AA) per V, D (for IGH), and J genes (in pink) [24]. Clonal expression is visualized with histograms, which represent the number of sequences assigned to IMGT clonotypes (AA) per V (in green), D (in red), and J (in yellow) genes [24]. Values are normalized, respectively, for 10,000 IMGT clonotypes (AA) to represent IG diversity immunoprofiles per V, D (for IGH), and J genes, and for 10,000 sequences assigned to IMGT clonotypes (AA) to represent IG expression immunoprofiles per V, D (for IGH), and J genes [24,70]. These normalized values allow comparative analysis studies performed with the same IMGT/HighV-QUEST standards [24,70].

### 3.3. IMGT/DomainGapAlign

#### 3.3.1. IMGT/DomainGapAlign Tool

IMGT/DomainGapAlign [10,25,26] is the IMGT^®^ online tool for the analysis of amino acid sequences and 2D structures of domains (e.g., V and C for IG) (Table 5). It analyzes V, C, and G domain amino acid sequences by comparison with the IMGT/DomainSeq reference directory that comprises sets for the different domain types (84). IMGT/DomainGapAlign functionalities include: introduction of “IMGT gaps” in the user amino acid sequences; alignments and identification of the genes and alleles by comparison with the closest domain(s) or region(s); delimitation of the domain(s) (e.g., V-DOMAIN or C-DOMAIN for IG) in the user sequence; delimitations of the regions of the IG and TR V-DOMAIN, in the user sequence; description of the amino acid (AA) changes, and IMGT Collier de Perles.

#### 3.3.2. IMGT/DomainSeq Reference Directory

The IMGT/DomainSeq reference directory [2] comprises the IMGT reference sequences (AA), against which IMGT/DomainGapAlign is running. It includes AA sequences (translation) from the IMGT Repertoire [2] and from IMGT/GENE-DB [8]. Sets are organized per domain (V, C, and G) (84). However, owing to the particularities of the IG and TR V-DOMAIN synthesis [3,4] there is no V-DOMAIN in the V sets of the IMGT/DomainSeq reference directory [2]. Instead, the directory comprises the translation of the IG and TR germline V and J genes (V-REGION and J-REGION, respectively). The IMGT/DomainSeq reference directory provides the IMGT^®^ “gene” and “allele” names. Data are comprehensive for human and mouse IG (and also TR) whereas for other species and other IgSF and MhSF they are added progressively. The IMGT/DomainSeq reference directory comprises AA sequences of domains, or of core regions for V and J, of functional (F), ORF (open reading frame) and in frame pseudogene (P) genes. As IMGT^®^ alleles are characterized at the nucleotide level, identical sequences at the amino acid level may therefore correspond to different alleles, in the IMGT/DomainSeq reference directory. The sequences can be displayed by querying IMGT/DomainDisplay [1].

## 4. IMGT^®^ Databases for IG V-DOMAIN and C-DOMAIN Analysis

### 4.1. IMGT/3Dstructure-DB

#### 4.1.1. IMGT/3Dstructure-DB card

IMGT/3Dstructure-DB [9,10,11]; the IMGT^®^ structure database; provides IMGT^®^ annotation and contact analysis of IG 3D structures; and paratope/epitope description of IG/antigen complexes (Table 5). There is one “IMGT/3Dstructure-DB card” per IMGT/3Dstructure-DB entry and this card provides access to all data related to that entry. The “PDB code” (4 letters and/or numbers; e.g., 1n0x) is used as “IMGT entry ID” for the 3D structures obtained from the Research Collaboratory for Structural Bioinformatics (RCSB) Protein Data Bank (PDB) [98]. The IMGT/3Dstructure-DB card provides eight search/display options: “Chain details”; “Contact analysis”; “Paratope and epitope”; “3D visualization Jmol or QuickPDB”; “Renumbered IMGT files”; “IMGT numbering comparison”; “References and links”; and “Printable card” [9,10,11].

#### 4.1.2. IMGT Chain and Domain Annotation

**Figure 5:**
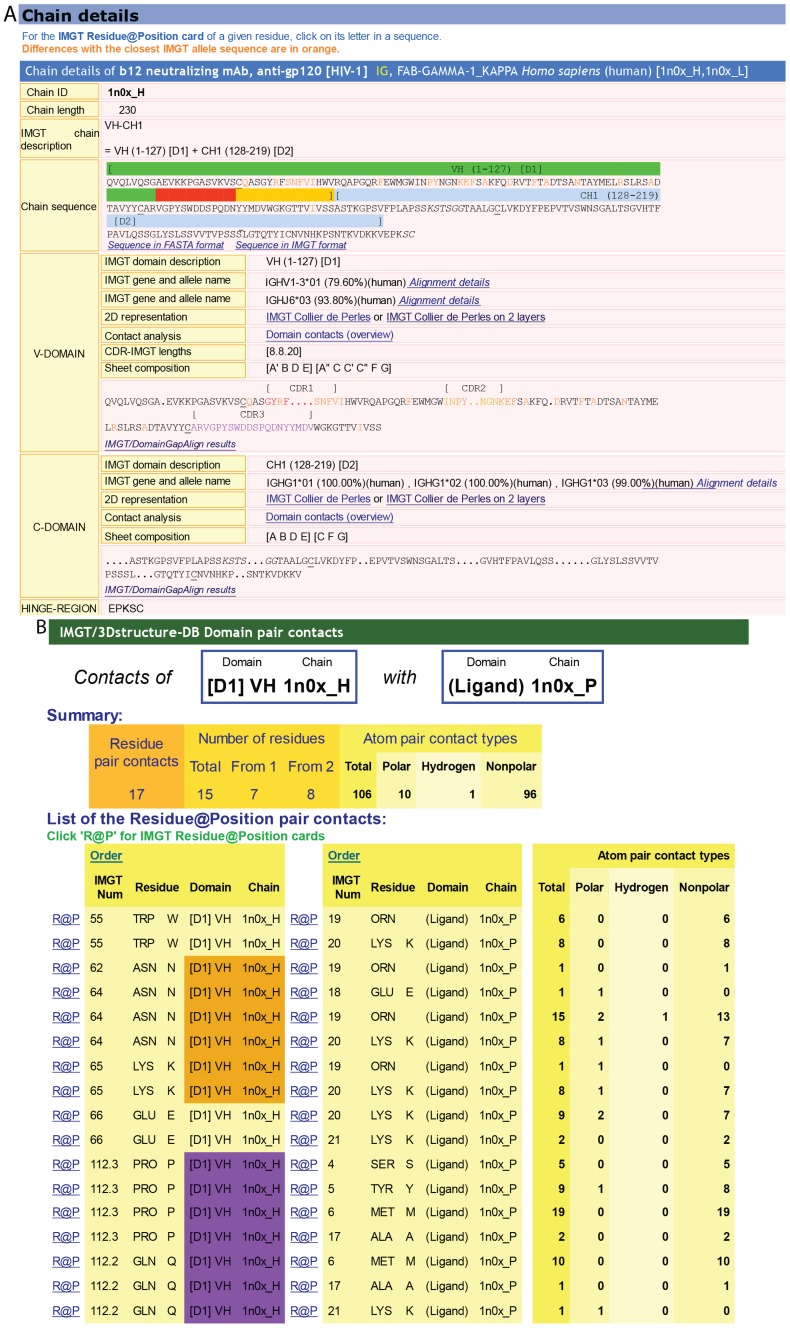

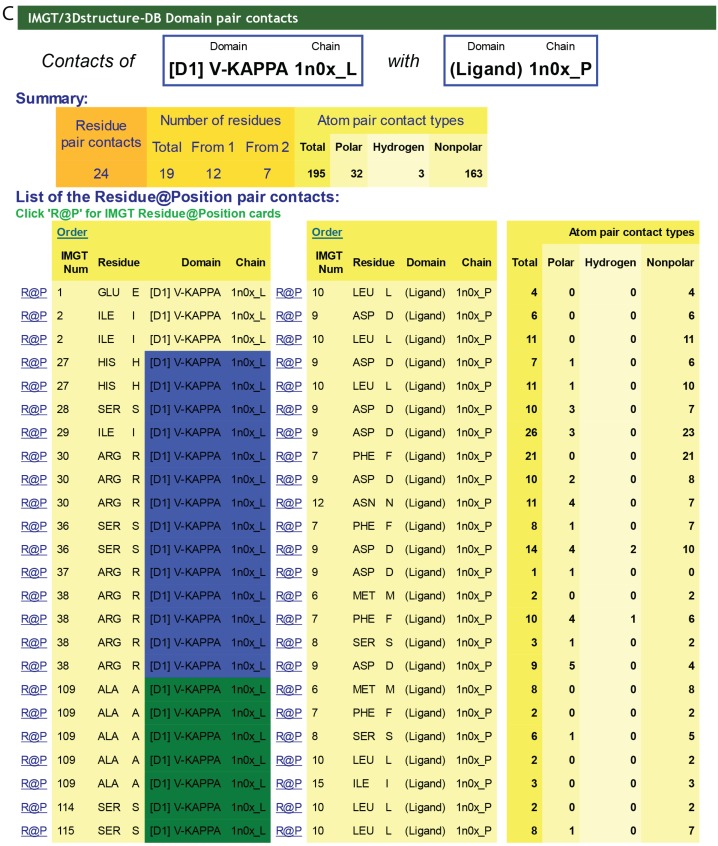
IMGT/3Dstructure-DB. (**A**) IMGT/3Dstructure-DB card. The “IMGT/3Dstructure-DB card” is available for each entry of the database. The “Chain details” shows, first, information on the chain (Chain ID, Chain length, IMGT chain description, Chain sequence), then a detailed description of each domain of the chain. The description of the V-DOMAIN (VH) and C-DOMAIN (CH1) of the VH-CH1 chain (1n0x_H) of the b12 Fab is shown. A similar result display interface is provided in IMGT/2Dstructure-DB cards but without “Contact analysis” (and without hydrogen bonds in IMGT Collier de Perles on 2 layers); (**B**) IMGT/3Dstructure-DB Domain pair contacts between the “VH” and the “Ligand” (antigen, Ag) of an IG/Ag complex. The VH is from the VH-CH1 chain (1n0x_H) of the b12 Fab and the ligand is a synthetic peptide (1n0x_P). The VH is in contact with the ligand by three AA of the CDR2-IMGT (orange online) (N62, N64 and K65) and two AA of the CDR3-IMGT (purple online) (P112.3 and Q112.2). The two AA which interact with the ligand but do not belong to the CDR-IMGT are the anchors W55 and E66. These contacts are not unexpected given by the small size (peptide) of the ligand; (**C**) IMGT/3Dstructure-DB Domain pair contacts between the “V-KAPPA” and the “Ligand” (Ag) of an IG/Ag complex. The V-KAPPA is from the L-KAPPA chain (1n0x_L) of the b12 Fab and the ligand is the peptide (1n0x_P) as in (**B**). The V-KAPPA is in contact with the ligand by seven AA of the CDR1-IMGT (blue online) (H27, S28, I29, R30, S36, R37 and R38) and three AA of the CDR3-IMGT (greenblue online) (A109, S114 and S115). “Polar”, “Hydrogen bond” and “Nonpolar” are selected by default in “Atom pair contact types” options at the bottom of the page (not shown). The user can also choose to display these contacts by “Atom pair contact categories” (BB), (SS), (BS) and (SB). Clicking on R@P gives access to the IMGT Residue@Position card. The IG/Ag complex structure is 1n0x from IMGT/3Dstructure-DB [1,9,10,11].

The “Chain details” section comprises information first on the chain itself, then per domain [9,10,11]. Chain and domain annotation includes the IMGT gene and allele names (CLASSIFICATION), region and domain delimitations (DESCRIPTION) and domain AA positions according to the IMGT unique numbering (NUMEROTATION) [60,61,62,63,64,65] (Figure 5A). The closest IMGT^®^ genes and alleles (found expressed in each domain of a chain) are identified with the integrated IMGT/DomainGapAlign [10,25,26], which aligns the amino acid sequences of the 3D structures with the IMGT/DomainSeq reference directory.

#### 4.1.3. Contact Analysis

“Contact analysis” gives access to a table with the different “Domain pair contacts” of the 3D structure (this table is also accessed from “Chain details” by clicking on “Domain contact (overview)’). “Domain pair contacts” refer to contacts between a pair of domains or between a domain and a ligand. Clicking on “DomPair” gives access to the contacts between amino acids for a given “Domain pair contacts”. Contacts between VH and the Ligand (antigen, Ag) and the V-KAPPA and the Ligand (Ag) of an IG/Ag complex are shown in Figure 5B,C, respectively. These contact analysis representations are important as they demonstrate that most, if not all, contacts with the ligand involve the amino acids of the CDR-IMGT. They definitively confirm the CDR-IMGT delimitations as the official reference standards [65,69,84].

In IMGT/3Dstructure-DB, all contacts are described as atom pair contacts. Atom pair contacts are obtained by a local program in which atoms are considered to be in contact when no water molecule can take place between them [9,10]. Atom pair contacts are provided by atom contact types (noncovalent, polar, hydrogen bond, nonpolar, covalent, disulfide) and/or atom contact categories (BB, backbone/backbone; SS, side chain/side chain; BS, backbone/side chain; SB, side chain/backbone) [9,10].

Clicking on “R@P” gives access to the IMGT identity card of a given residue (usually an amino acid) at a given position or Residue@Position. The IMGT R@P card can also be accessed from the amino acid sequences of the IMGT/3Dstructure-DB card or from the IMGT Collier de Perles, by clicking on one amino acid. In an IMGT R@P card, the Residue@Position is defined by the IMGT position numbering in a domain, or if not characterized, in the chain, the amino acid name (3-letter and, between parentheses, 1-letter abbreviations), the IMGT domain description, and the IMGT chain ID, e.g., “103—TYR (Y)—VH—1hzh_H” [9,10,11]. The IMGT R@P card includes: (1) general information (PDB file numbering, IMGT file numbering, residue full name and formula); (2) structural information “IMGT LocalStructure@Position” (secondary structure, Phi and Psi angles (in degrees), and accessible surface area (ASA) (in square angstrom)); and (3) detailed contact analysis with amino acids of other domains [9,10,11].

#### 4.1.4. Paratope and Epitope

In an IG/Ag complex, the amino acids in contact at the interface between the IG and the Ag constitute the paratope on the IG V-DOMAIN surface and the epitope on the Ag surface. For IG/Ag, the paratope and epitope are displayed in Contact analysis (Figure 5B,C), but for each V domain separately. Clicking on the “Paratope and epitope” tag (displayed in the IMGT/3Dstructure-DB card, only if relevant), gives access to “IMGT paratope and epitope details”, which are described in a standardized way. Each amino acid which belongs to the paratope is defined by its position in an IG V-DOMAIN. Each amino acid that belongs to the epitope is defined by its position in the chain in the 3D structure or, if the antigen belongs to an IgSF or MhSF protein and if the epitope is part of a characterized V, C, or G domain, by its position in the domain according to the IMGT unique numbering.

#### 4.1.5. Renumbered Flat File and IMGT Numbering Comparison

“Renumbered IMGT file” allows viewing (or downloading) of an IMGT coordinate file renumbered according to the IMGT unique numbering, and with added IMGT specific information on chains and domains (added in the “REMARK 410” lines (blue online) and identical to the “Chain details” annotation).

“IMGT numbering comparison” provides, per domain, the IMGT DOMAIN numbering by comparison with the PDB numbering and the residue (3-letter and 1-letter names), which allows standardized IMGT representations using generic tools.

#### 4.1.6. IMGT/3Dstructure-DB Associated Tools

Tools associated with IMGT/3Dstructure-DB include IMGT/StructuralQuery [9] and IMGT/DomainSuperimpose, available online. IMGT/StructuralQuery allows retrieval of the IMGT/3Dstructure-DB entries, based on specific structural characteristics of the intramolecular interactions: phi and psi angles, accessible surface area, type of atom contacts, distance in angstrom between amino acids, IMGT Residue@Position contacts, and, for V-DOMAIN, CDR-IMGT length or pattern [9]. IMGT/DomainSuperimpose allows superimposing of the 3D structures of two domains from IMGT/3Dstructure-DB.

### 4.2. IMGT/2Dstructure-DB

IMGT/2Dstructure-DB was created as an extension of IMGT/3Dstructure-DB [9,10,11] to describe and analyze amino acid sequences of chains and domains for which no 3D structures were available (Table 5). IMGT/2Dstructure-DB uses the IMGT/3Dstructure-DB informatics frame and interface, which allow one to analyze, manage and query IG (and also TR and MH, as well as other IgSF and MhSF) and engineered proteins (FPIA, CPCA) as polymeric receptors made of several chains, in contrast to the IMGT/LIGM-DB sequence database that analyzes and manages sequences individually [7]. The amino acid sequences are analysed with the IMGT^®^ criteria of standardized identification [57], description [58], nomenclature [59] and numerotation [60,61,62,63,64,65].

The current IMGT/2Dstructure-DB entries include amino acid sequences of antibodies from Kabat [95] (those for which there were no available nucleotide sequences) and amino acid sequences of mAb and FPIA from the WHO-INN programme [12,48,49]. Queries can be made on an individual entry using the “Entry ID” or the “Molecule name”. The same query interface is used for IMGT/2Dstructure-DB and IMGT/3Dstructure-DB. Thus a “trastuzumab’ query in “Molecule name” allows retrieval of six results: two INN (“trastuzumab” and “trastuzumab emtansine”) from IMGT/2Dstructure-DB and four 3D structures from IMGT/3Dstructure-DB. For mAb and FPIA results, INN sequences represent the reference sequences [12,48,49] as sequences of the 3D structures may have been engineered or may contain experimental errors.

The IMGT/2Dstructure-DB cards provide standardized IMGT information on IG chains and domains and IMGT Colliers de Perles on one or two layers, in a format identical to that provided for the sequence analysis in IMGT/3Dstructure-DB; however, the information on experimental structural data (hydrogen bonds in IMGT Collier de Perles on two layers, Contact analysis) is only available in the corresponding IMGT/3Dstructure-DB cards if the antibodies have been crystallized.

## 5. IMGT^®^ IG V-DOMAIN and C-DOMAIN Analysis for Antibody Humanization and Engineering

### 5.1. CDR-IMGT Delimitation for Grafting

The objective of antibody humanization is to graft at the DNA level the CDR of an antibody V domain, from mouse (or other species) and of a given specificity, onto a human V domain framework, thus preserving the specificity of the original (murine or other species) antibody while decreasing its immunogenicity [99]. IMGT/DomainGapAlign [10,25,26] is the reference tool for antibody humanization design based on CDR grafting. Indeed, it precisely defines the CDR-IMGT to be grafted and helps in selecting the most appropriate human FR-IMGT by providing the alignment of the amino acid sequences between the mouse (or other species) and the closest human V-DOMAIN.

Analyses performed on humanized therapeutic antibodies underline the importance of a correct delimitation of the CDR and FR. As an example, two amino acid changes were required in the first version of the humanized VH of alemtuzumab, in order to restore the specificity and affinity of the original rat antibody. The positions of these amino acid changes (S28 > F and S35 > F) are now known to be located in the CDR1-IMGT and should have been directly grafted, but at the time of this mAb humanization they were considered as belonging to the FR according to the Kabat numbering [95]. In contrast, positions 66–74 were, at the same time, considered as belonging to the CDR according to the Kabat numbering, whereas they clearly belong to the FR2-IMGT and the corresponding sequence should have been “human” instead of being grafted from the “rat” sequence (IMGT^®^ [1], The IMGT Biotechnology page > Antibody humanization > Alemtuzumab).

### 5.2. Evaluation of the Degree of “Humanization” of an IG V Sequence

IMGT/DomaingapAlign is used to evaluate the degree of “humanization” of an IG V sequence, either obtained from a species other than human (e.g., mouse or rat), or obtained from engineered human sequences (e.g., selected from combinatorial library or mutated). IMGT/DomaingapAlign provides an objective assessment of the degree of humanization of the user sequence, based on sequence alignments, independently on the source of the starting sequence (e.g., species) and independently on the experimental methodology that was used with the objective of humanizing it. A query of the user sequence against “V” of “any” species will display “*Homo sapiens*” IG V genes at the top of the results, in the case of a successfully “humanized” V. In contrast, the query will display V genes of species other than *Homo sapiens* for an unsuccessful humanization: in that case the V gene is “non-human” and the IG chain to which it belongs is “chimeric”.

### 5.3. IGHG1 Alleles and G1m Allotypes

Allotypes are polymorphic markers of an IG subclass that correspond to amino acid changes and are detected serologically by antibody reagents [77]. In therapeutic antibodies (human, humanized or chimeric) [12], allotypes may represent potential immunogenic residues [76], as demonstrated by the presence of antibodies in individuals immunized against these allotypes [77]. The allotypes of the human heavy gamma chains of the IgG are designated as Gm (for gamma marker).

The allotypes G1m, G2m and G3m are carried by the constant region of the gamma1, gamma2 and gamma3 chains, encoded by the IGHG1, IGHG2 and IGHG3 genes, respectively [77]. The gamma1 chains express different combinations of G1m allotypes or G1m alleles: G1m3, G1m3,1, G1m17,1, and G1m17,1,2 (Table 6). The C region of the G1m3,1, G1m17,1 and G1m17,1,2 chains differ from that of the G1m3 chains by two, three and four amino acids, respectively [77]. Two additional G1m alleles (G1m17,1,28 and G1m17,1,27,28) have been identified by serology in the Negroid populations, whereas another allele (G1m17,1,27) was deduced from a sequence with the AA change expected for the Gm27 allotype [77]. The correspondence between the G1m alleles and IGHG1 alleles is shown in Table 6.

In the IGHG1 CH1, the lysine at position 120 (K120) in strand G corresponds to the G1m17 allotype [77] (Figure 4D). The isoleucine I103 (strand F) is specific of the gamma1 chain isotype. If an arginine is expressed at position 120 (R120), the simultaneous presence of R120 and I103 corresponds to the expression of the G1m3 allotype [77]. For the gamma3 and gamma4 isotypes (which also have R120 but T in 103), R120 only corresponds to the expression of the nG1m17 isoallotype (an isoallotype or nGm is detected by antibody reagents that identify this marker as an allotype in one IgG subclass and as an isotype for other subclasses) [77].

**Table 6:** Correspondence between the IGHG1 alleles and G1m alleles.

IGHG1 Alleles	G1m Alleles ^a^			IMGT Amino acid Positions ^b^						Populations [77]
	allotypes		Isoallotypes ^c^	CH1			CH3				
				103		120	12	14	110		
						G1m17/nG1m1	G1m1/nG1m1		G1m2/-		
				G1m3 ^d^							
IGHG1*01 ^e^, IGHG1*02 ^e^	G1m17,1			I		K	D	L	A	Caucasoid Negroid Mongoloid	
IGHG1*04	G1m17,1,27										
IGHG1*05p	G1m17,1,28									Negroid	
IGHG1*06p	G1m17,1,27,28									Negroid	
IGHG1*03	G1m3		*nG1m1, nG1m17*	I		R	E	M	A	Caucasoid	
IGHG1*07p ^f^	G1m17,1,2			I		K	D	L	G	Caucasoid Mongoloid	
IGHG1*08p ^f^	G1m3,1		*nG1m17*	I		R	D	L	A	Mongoloid	

^a^ In Negroid populations, the G1m17,1 allele frequently includes G1m27 and G1m28, leading to two additional G1m alleles, G1m17,1,27 and G1m17,1,27,28, as demonstrated serologically [77]. They were assigned to IGHG1*05p and IGHG1*06p, respectively, following the recent sequencing of IGHG1*04 (IMGT/GENE-DB [8]) [77]. The letter “p” indicates that these alleles have not yet been sequenced at the nucleotide level, and therefore are not shown in IMGT Repertoire > Alignments of alleles: *Homo sapiens* IGHG1 [2]. ^b^ Amino acids corresponding to G1m allotypes are shown in bold. Amino acid changes and codons for G1m27 (CH3 Ileu I101) and G1m28 (most probably CH3 Arg R115, Tyr Y116) are not shown. ^c^ The nG1m1 and nG1m17 isoallotypes present on the Gm1-negative and Gm17-negative gamma1 chains (and on other gamma chains) are shown in italics. ^d^ The presence of R120 is detected by anti-nG1m17 antibodies whereas the simultaneous presence of I103 and R120 in the gamma1 chains is detected by anti-Gm3 antibodies [77]. ^e^ The IGHG1*01 and IGHG1*02 alleles only differ at the nucleotide level (codon 85.1 in CH2). ^f^ IGHG1*07p and IGHG1*08p amino acids are expected [77] but not yet sequenced at the nucleotide level and therefore the IGHG1*07p and IGHG1*08p alleles are not shown in IMGT Repertoire, Alignments of alleles: *Homo sapiens* IGHG1 [1].

In the IGHG1 CH3, the aspartate D12 and leucine L14 (strand A) correspond to G1m1, whereas glutamate E12 and methionine M14 correspond to the nG1m1 isoallotype [77] (Table 6). A glycine at position 110 corresponds to G1m2, whereas an alanine does not correspond to any allotype (G1m2-negative chain). Therapeutic antibodies are most frequently of the IgG1 isotype, and to avoid a potential immunogenicity, the constant region of the gamma1 chains are often engineered to replace the G1m3 allotype by the less immunogenic G1m17 (CH1 R120 > K) (G1m17 is more extensively found in different populations) [77].

### 5.4. IGHG N-Linked Glycosylation Site CH2 N84.4

A N-linked glycosylation site is present in the CH2 domain of the constant region of the human IG heavy chains of the four IgG isotypes. The N-linked glycosylation site belongs to the classical N-glycosylation motif N-X-S/T (where N is asparagine, X any amino acid except proline, S serine, T threonine) and is defined as CH2 N84.4. As shown in the IMGT Collier de Perles, this asparagine is localized at the DE turn. The IMGT unique numbering has the advantage of identifying the C domain (here, CH2) and, in the domain, the amino acid and its localization (here, N84.4) which can be visualized in the IMGT Collier de Perles and correlated with the 3D structure [70,84,85].

## 6. Conclusions

IMGT-ONTOLOGY and the IMGT^®^ information system, which are at the origin of immunoinformatics [45], have provided the concepts, the knowledge environment and the informatics frame for a standardized and integrated analysis of the IG, from gene to structure and function. IG repertoire analysis, therapeutic antibody engineering and humanization, paratope/epitope characterization, immunotherapy represent major current fields of immunoinformatics at the forefront of basic, pharmaceutical and clinical research owing to major methodological and medical advances.

The IMGT^®^ standards for IG are used in clinical applications. Thus, IMGT/V-QUEST is frequently used by clinicians for the analysis of IG somatic hypermutations in leukemia, lymphoma and myeloma, and more particularly in chronic lymphocytic leukemia (CLL) [16,72,73,74,75] in which the percentage of mutations of the rearranged IGHV gene in the VH of the leukemic clone has a prognostic value for the patients. For this evaluation, IMGT/V-QUEST is the standard recommended by the European Research Initiative on CLL (ERIC) for comparative analysis between laboratories [72]. The sequences of the V-(D)-J junctions determined by IMGT/JunctionAnalysis [19,20] are also used in the characterization of stereotypic patterns in CLL [73,74] and for the synthesis of probes specific of the junction for the detection and follow-up of minimal residual diseases (MRD) in leukemias and lymphomas. A new era is opening in hemato-oncology with the use of NGS for analysis of the clonality and MRD identification, making IMGT^®^ standards use more needed as ever. More generally, the IMGT/HighV-QUEST web portal is a paradigm for identification of IMGT clonotype diversity and expression in NGS immune repertoire analysis of the adaptive immune response in infectious diseases, in vaccination, and for next generation repertoire immunoprofiling [24].

The therapeutic monoclonal antibody engineering field represents the most promising potential in medicine. A standardized analysis of IG genomic and expressed sequences, structures and interactions is crucial for a better molecular understanding and comparison of the mAb specificity, affinity, half-life, Fc effector properties and potential immunogenicity. IMGT-ONTOLOGY concepts have become a necessity for IG loci description of newly sequenced genomes, antibody structure/function characterization, antibody engineering (single chain Fragment variable (scFv), phage displays, combinatorial libraries) and antibody humanization (chimeric, humanized and human antibodies) [33,40,82,83,84,85,86]. IMGT^®^ standardization allows repertoire analysis and antibody humanization studies to move to novel high-throughput methodologies with the same high-quality criteria. The CDR-IMGT lengths are now required for mAb INN applications and are included in the WHO-INN definitions [49], bringing a new level of standardized information in the comparative analysis of therapeutic antibodies.

## Availability and Citation

Authors who use IMGT^®^ databases and tools are encouraged to cite this article and to quote the IMGT^®^ Home page, http://www.imgt.org. Online access to IMGT^®^ databases and tools are freely available for academics and under licences and contracts for companies.
